# Evaluation of SARS-CoV-2 Antibodies and the Impact of COVID-19 on the HIV Care Continuum, Economic Security, Risky Health Behaviors, and Mental Health Among HIV-Infected Individuals in Vietnam

**DOI:** 10.1007/s10461-021-03464-w

**Published:** 2021-09-27

**Authors:** Shoko Matsumoto, Moeko Nagai, Dieu An Dang Luong, Hoai Dung Thi Nguyen, Dung Thi Nguyen, Trang Van Dinh, Giang Van Tran, Junko Tanuma, Thach Ngoc Pham, Shinichi Oka

**Affiliations:** 1 AIDS Clinical Center, National Center for Global Health and Medicine, 1-21-1, Toyama, Shinjuku, Tokyo, 162-8655 Japan; 2 National Hospital for Tropical Diseases, Hanoi, Vietnam; 3 Hanoi Medical University, Hanoi, Vietnam

**Keywords:** COVID-19, SARS-CoV-2, Antibody, HIV, Mental health

## Abstract

Coronavirus disease 2019 (COVID-19) and associated social responses may uniquely affect people living with HIV (PLHIV). SARS-CoV-2 antibody testing and a cross-sectional survey on COVID-19’s socio-behavioral impacts were conducted among a large PLHIV cohort in Hanoi, Vietnam. We examined anonymous antibody test results for 1243 PLHIV (99.8%) from whom plasma was obtained and completed surveys were collected in June/July 2020, just after the end of the first COVID-19 outbreak and nationwide lockdown. Three participants (0.2%) tested positive for anti-SARS-CoV-2 IgG antibodies. HIV treatment was generally maintained without antiretroviral therapy interruption, but COVID-19 had substantial impacts on economic security and risky health behaviors among PLHIV, which may have amplified psychological stress. These findings highlight the need for continuous monitoring of COVID-19’s impacts on PLHIV and for efforts to mitigate these impacts.

**Supplementary Information:**

The online version contains supplementary material available at 10.1007/s10461-021-03464-w.

## Introduction

Since the first case of coronavirus disease 2019 (COVID-19) was reported in Wuhan, China, in December 2019, COVID-19 has spread rapidly throughout the world, with over 115 million confirmed cases and over 2.5 million deaths as of March 4, 2021 [1]. Approximately 38 million people living with HIV (PLHIV) [2] are at risk of infection with severe acute respiratory syndrome coronavirus 2 (SARS-CoV-2) and contraction of COVID-19. However, it is unclear whether HIV infection is associated with SARS-CoV-2 infection, disease progression, or death. Some previous studies have indicated that PLHIV are affected by similar features of disease risk and progression as HIV-uninfected patients [3–8]. Other studies have reported that the risk of serious symptoms and death may be higher among PLHIV, especially those who are not taking antiretroviral therapy (ART), those who are immunocompromised, and those who are suffering from chronic conditions (i.e., renal dysfunction) [9–11].

In addition to complications of the disease itself, COVID-19 potentially affects PLHIV in multiple ways. One of the greatest concerns is the disruption of the HIV care continuum [12–15]. For those who are taking ART, the implementation of city lockdowns or traffic controls may hinder regular hospital visits and ART continuation. Discontinuation of ART could lead to an increase in HIV viral load, the development of opportunistic infection, and death [16, 17]. Another concern regarding PLHIV is that the COVID-19 pandemic may accelerate HIV transmission by affecting risky health behaviors including substance use and risky sexual behaviors. For example, restricted mobility and psychological stress because of social distancing and quarantine, perhaps with a romantic partner, may promote engaging in risky behavior [7]. Moreover, both COVID-19 and HIV infection disproportionately affect marginalized groups, including racial/ethnic and sexual minorities, as well as those living in poverty [18–20]. Thus, COVID-19 may exacerbate social isolation, unemployment, and poverty in the population with HIV. Finally, considering the high prevalence of depression in this population [21, 22], these individuals’ mental health may be negatively affected by psychological pressure to continue ART, changes in health behaviors during the pandemic, and negative economic and psychosocial consequences.

The above-mentioned concerns are also relevant for the estimated 230,000 PLHIV in Vietnam [23]. Vietnam is among the countries known to be successfully controlling the COVID-19 pandemic, with 2482 infected cases and 35 deaths confirmed as of March 4, 2021 [1]. After the first case of COVID-19 in the country was found in January 2020, Vietnam experienced three outbreaks of SARS-CoV-2 infection—the first from January to April 2020, the second from July to September 2020 (the provinces of Da Nang and Quang Nam were the most affected), and the third beginning in January 2021 (on-going as of March 2021, Hai Duong Province has been the most affected). The government of Vietnam has taken strong leadership under the principle of “protecting people’s health first” and applied strict measures to respond rapidly to these outbreaks [24]. In response to the first outbreak, the country entered a nationwide lockdown beginning on April 1, 2020, which substantially affected people’s everyday lives. During this lockdown, some infectious disease departments in hospitals that usually provide HIV services allocated medical personnel and resources to treat people who were infected with SARS-CoV-2. In Hanoi, Bach Mai Hospital and the National Hospital for Tropical Diseases (NHTD) in Giai Phong District, the two largest central-level HIV outpatient clinics, which are located on the same premises, were locked down for 14 days in March and April 2020 because Bach Mai Hospital had a nosocomial cluster of confirmed COVID-19 cases. Patients with HIV often go to hospitals located in provinces far from their residences to avoid their HIV status being disclosed where they live. The restrictions on mobility across provinces thus prevented some patients with HIV from seeing their family doctors. Furthermore, by December 2020, the COVID-19 pandemic had caused 32.1 million workers in Vietnam aged 15 years or older to either lose their jobs or have their working hours reduced [25]. Considering that PLHIV in Vietnam experience higher rates of poverty and unemployment compared with those without HIV [26], the economic impact of COVID-19 may be particularly serious among PLHIV.

The characteristics of both the HIV epidemic and COVID-19 response efforts vary across countries and in different socio-cultural contexts. Understanding how PLHIV are uniquely affected by the COVID-19 pandemic and the associated social responses is of high public health importance to help policy makers and stakeholders implement appropriate strategies to mitigate the negative impacts. Therefore, this study aimed to evaluate the incidence of SARS-CoV-2, behaviors to prevent COVID-19, and the impacts of COVID-19 on the HIV continuum of care, economic security, and risky health behaviors among Vietnamese PLHIV during the first COVID-19 outbreak in Vietnam. We also investigated how the impact of COVID-19 affected HIV viral load and mental health after the first outbreak.

## Methods

### Study Design and Study Subjects

We conducted a self-administered questionnaire survey and SARS-CoV-2 antibody testing among a hospital-based cohort of PLHIV receiving ART (aged ≥ 18 years). This cohort (the “Hanoi cohort”) was established in 2007 at the HIV outpatient clinic at the NHTD [27, 28]. Prospective clinical data (e.g., ART history and HIV viral load) were obtained every 6 months under the Japan Initiative for Global Research Network on Infectious Diseases (2006–2018) and Science and Technology Research Partnership for Sustainable Development (SATREPS, 2019–present) programs. Both the questionnaire survey and the blood sampling for the SARS-CoV-2 antibody test were carried out in June and July 2020 in addition to the regular SATREPS data collection. These data were collected just after the first COVID-19 outbreak had ended—the lockdown was lifted in Hanoi on April 23, 2020, and all the medical services at the NHTD returned to normal on May 1, 2020. No vaccines were available at that time in Vietnam. The SARS-CoV-2 antibody testing was conducted in January 2021 using the stored plasma samples.

The Hanoi cohort participants usually received ART at the NHTD every 1–3 months, depending on their health condition and ART regimen. However, during the hospital lockdown in the first outbreak of COVID-19, those who were facing medicine stockouts were referred to other HIV outpatient clinics to continue ART.

All Hanoi cohort participants were invited to complete the survey and to have a blood sample collected for the antibody test during their first regular consultation after the hospital lockdown was lifted. Individuals who provided informed consent participated in the survey on the same day as their consultation.

### Measurements

#### Incidence of SARS-CoV-2 Infection

The incidence of SARS-CoV-2 infection was investigated both by asking whether the participant had been diagnosed with SARS-CoV-2 infection by polymerase chain reaction test or quarantined as a close contact of another person with such a diagnosis in the questionnaire survey and by laboratory-based immunoassay to detect anti-SARS-CoV-2 IgG antibodies.

IgG antibodies represent relatively long-lasting antibodies against the virus, and a positive IgG antibody result indicates past infection with the virus [29]. We collected venous blood samples to separate out plasma. All plasma samples were stored at − 80 °C before testing. IgG antibodies against SARS-CoV-2 in the plasma were qualitatively analyzed using a Chemiluminescent Microparticle Immunoassay kit (6R86 SARS-CoV-2 IgG Reagent Kit, Abbott Laboratories Inc. Abbott Park, IL, USA). The tests were conducted on an automated immunoassay system (ARCHITECT i2000, Abbott Laboratories Inc.).

#### Behaviors to Prevent COVID-19

Behaviors to prevent COVID-19 were evaluated in the questionnaire by asking whether participants practiced any protective measures against COVID-19 and about frequency of social contact with others during the first COVID-19 outbreak. Social contact included non-face-to-face contacts through email, telephone, and social networking services. Those who responded affirmatively to the initial question about practicing protective measures against COVID-19 were further asked about the specific measures. Response options were *social distancing*, *hand washing*, *wearing a mask*, and *other*. Participants were instructed to select as many items as were applicable. The possible responses for frequency of social contact with others during the outbreak compared with before the outbreak were *no change*, *decreased*, and *increased*.

#### HIV Continuum of Care

In the questionnaire, access to ART during the first COVID-19 outbreak was assessed by asking whether the participants had ever had to go to a hospital other than the NHTD to receive ART because of the first COVID-19 outbreak, and discontinuation of ART because of the outbreak was assessed by asking whether they had ever stopped taking ART because the outbreak caused them to run out of medicine. Those who temporarily received ART at another hospital were also asked whether their ART regimen was changed at this other hospital. Those who discontinued ART were also asked about the duration of the ART interruption. Additionally, social support for continuing HIV care and treatment during the first COVID-19 outbreak was assessed. Those who received support were asked about the specific sources of support. Possible response options were *government agencies including the Vietnam Administration of HIV/AIDS Control*, *NHTD health care staff members*, *other PLHIV*, and *other*. Participants were instructed to select as many items as were applicable.

Information on HIV viral load and loss to follow-up before and after the first outbreak of COVID-19 was also obtained from the SATREPS. HIV viral load in October or November 2019 was used for the result before the outbreak. HIV viral load in June or July 2020 was used for the result after the outbreak. Loss to follow-up was defined as a participant missing a scheduled visit without a known reason. The number of participants who were lost to follow-up was counted during the 6 months before and the 6 months after the outbreak, from June 1, 2019, to November 30, 2019, and from December 1, 2019, to May 31, 2020, respectively.

#### Economic Impact

The economic impact of the COVID-19 pandemic was evaluated in the questionnaire by asking whether the participants’ employment status or household income had changed as a result of the first COVID-19 outbreak. Those who responded affirmatively to the employment status question were asked to select *lost jobs*, *started working*, or *changed jobs*; those responding affirmatively to the household income question were asked to report their actual household income before and after the outbreak. Depending on the difference between these figures, the change in household income was categorized as no change, increased, or decreased.

#### Risky Health Behaviors

Changes in the following three risky health behaviors before and after the first COVID-19 outbreak were also assessed in the questionnaire: alcohol intake, drug use (e.g., heroin, cocaine, marijuana, LSD), and risky sexual behaviors (i.e., having sex without a condom and having multiple sex partners). Participants were asked whether there were any changes in their alcohol consumption, amount of drug use, frequency of using a condom during sex, or number of sex partners before and after the first COVID-19 outbreak. The possible responses were *no change*, *decreased*, and *increased*. For the items on alcohol intake and drug use, *non-drinker* and *non-drug user* were also added to the response categories.

#### Mental Health

The Vietnamese version of the Depression, Anxiety, and Stress Scale-21 (DASS-21) [30] was used to assess the mental health status of the participants. The DASS-21 is a set of three self-report scales measuring the negative emotional states of depression, anxiety, and stress. This scale was developed using an Australian sample. Each subscale contains seven items answered on a four-point scale ranging from 0 (*did not apply to me at all*) to 3 (*applied to me very much* or *most of the time*). The total score for each subscale, calculated by multiplying the sum of the responses by two, ranges from 0 to 42. There was originally a set of cut-off scores for defining the level of severity (i.e., normal, mild, moderate, severe, and extremely severe) for the DASS-21. However, two previous studies assessing the validity of the Vietnamese version of the DASS-21 (DASS-21-V) among Vietnamese women and adolescents concluded that the scale was more suitable for use as a screening tool for symptoms of general distress than for detecting symptoms of depression, anxiety, and stress separately [31, 32]. Tran et al. [31] reported the optimal DASS-21-V cut-off score of 34 to detect common mental disorders including depression and anxiety, with a sensitivity of 79.1% and a specificity of 77.0%. Considering that the psychometric properties of mental health instruments can be strongly affected by language expression, cultural and social characteristics, and values and beliefs, we defined a total DASS-21-V score of 34 or higher as experiencing general distress.

#### Demographic Characteristics and HIV-Related Factors

Data on the following demographic characteristics and HIV-related factors were collected: sex, age, residence, marital status, educational attainment, employment before COVID-19, history of injection drug use (IDU), and duration on ART. Age was divided into quartiles: < 37 years, 37–41 years, 42–45 years, and ≥ 46 years. Residence was divided into two categories: Hanoi and other. Marital status was divided into three categories: married, unmarried, and widowed or divorced. Educational attainment was divided into three groups: low (never attended school, primary school, or junior high school), middle (high school), and high (vocational school/college or university). Employment before COVID-19 was categorized into five groups: full-time employee, part-time employee, self-employed full time, self-employed part time, and jobless and not working at all (including retirement). History of IDU was divided into two categories: ever used injection drugs and never used injection drugs. Duration on ART was divided into two categories using the median value: < 9 years and ≥ 9 years. For those with detected anti-SARS-CoV-2 IgG antibodies, data on body mass index, ART regimen at the time of testing, CD4 count (/µl) before the outbreak, and co-morbidities (i.e., hypertension, cardiovascular disease, diabetes, chronic kidney disease, chronic liver disease, and chronic respiratory disease) were also collected from the SATREPS.

### Statistical Analysis

Baseline characteristics were summarized for all participants. Then, we performed descriptive analyses to evaluate the incidence of SARS-CoV-2 infection, prevention measures, and the impact of COVID-19 on the HIV continuum of care, economic security, risky health behaviors, and mental health among the study participants. The incidence of loss to follow-up during the 6 months before and the 6 months after the COVID-19 outbreak was calculated by dividing the number of participants who were lost to follow-up during each 6-month follow-up period (i.e., June 1, 2019–November 30, 2019, and December 1, 2019–May 31, 2020) by the number of participants on the first day of each period. Logistic regression analyses were then performed to analyze the factors associated with having a detectable HIV viral load (> 20 copies/mL) and with general distress after the first COVID-19 outbreak. Crude odds ratios (ORs) were calculated.

All analyses were performed using SAS 9.4 software (SAS Institute Inc., Cary, NC, USA). All tests were two-sided, with the significance level set at 5%. Missing data were excluded from the analyses.

## Results

### Study Participants

Since October 2007, 1820 patients registered for the Hanoi cohort, and 1246 were still enrolled and underwent follow-up in June or July 2020. Of these patients, 1243 agreed to participate in the survey (response rate: 99.8%). Table 1 shows the respondents’ characteristics. In total, 57.7% of the participants were men. The median age (interquartile range) was 42 (37–46) years. More than half of the participants lived in provinces outside of Hanoi, 33.6% worked full time as employees before the first COVID-19 outbreak, 22.1% had a history of IDU, and nearly half had received ART for 9 years or longer.

**Table 1:** Characteristics of the study participants

	*n*	%
All participants	1243	100.0
Sex		
Male	717	57.7
Female	526	42.3
Age (years)		
Median (IQR)	42 (37–46)	
< 37	252	21.6
37–41	314	27.0
42–45	279	24.0
≥ 46	320	27.5
Residence		
Hanoi	574	46.2
Other	669	53.8
Marital status		
Married	818	65.8
Unmarried	86	6.9
Widowed or divorced	201	16.2
N/A	138	11.1
Educational attainment^a^		
Low	307	24.7
Middle	328	26.4
High	468	37.7
N/A	140	11.3
Employment before COVID-19		
Full-time employment as an employee	417	33.6
Part-time employment as an employee	74	6.0
Working full-time but not as an employee (self-employed)	262	21.1
Working part-time but not as an employee (self-employed)	175	14.1
Jobless and not working at all (including retirement)	176	14.2
N/A	139	11.2
History of IDU		
No (never used injection drugs)	968	77.9
Yes (ever used injection drugs)	275	22.1
Time since ART initiation (years)	108 (90,144)	
< 9	613	49.3
≥ 9	630	50.7

*IQR* interquartile range, *N/A* not available, *COVID-19* Coronavirus disease 2019, *IDU* injection drug use, *ART* antiretroviral therapy

^a^Educational attainment: low—never attended school, primary school, or junior high school; middle—high school; high—vocational school/college or university

### Incidence of SARS-CoV-2 Infection

There were no reports of SARS-CoV-2 infection or quarantine in the questionnaire responses. However, anti-SARS-CoV-2 IgG antibodies were detected in three participants (0.2%) (Table 2). Detailed information on these cases is described in Supplementary Table 1. All three were men and none had experienced symptoms of COVID-19; their ages ranged from 37 to 54 years. One had a history of IDU. HIV infection was well controlled for all three patients with an ART regimen of tenofovir disoproxil fumarate, lamivudine, and dolutegravir at the time of testing, and none of these patients reported comorbidities. All three patients reported that they practiced protective measures against COVID-19 (i.e., social distancing, hand washing, and wearing a mask), and two reported a decrease in social contact during the first outbreak.

**Table 2:** Incidence of SARS-CoV-2 infection

Variable	n	%
History of SARS-CoV-2 infection (self-reported)		
Never diagnosed or quarantined	1237	99.5
Diagnosed (by PCR test)	0	0.0
Quarantined	0	0.0
N/A	6	0.5
Anti-SARS-CoV-2 IgG antibodies		
Positive	3	0.2
Negative	1228	98.8
N/A	12	1.0

*SARS-CoV-2* severe acute respiratory syndrome coronavirus 2, *PCR* polymerase chain reaction, *N/A* not available

### Behaviors to Prevent COVID-19

Most participants (95.9%) engaged in one or more preventive measures against COVID-19; 90.0% wore a mask, 86.6% practiced hand washing, and 86.3% engaged in social distancing as preventive measures. Half of the participants (50.0%) reported a decrease in social contact with others during the first outbreak (Table 3).

**Table 3:** Descriptive analysis of the impacts of COVID-19: prevention, HIV continuum of care, economic security, and risky health behaviors

Variable	*n*	%
**Behaviors to prevent COVID-19**		
Protective behaviors against COVID-19		
Not practiced	26	2.1
Practiced	1192	95.9
N/A	25	2.0
Description of protective behaviors against COVID-19^a^ (among those who practiced protective behaviors)		
Social distancing	1073	86.3
Hand washing	1076	86.6
Wearing a mask	1119	90.0
Other	33	2.7
Social contact^b^ with other people during COVID-19		
No change	576	46.3
Decreased	622	50.0
Increased	10	0.8
N/A	35	2.8
**HIV continuum of care**		
Access to ART during COVID-19		
Continued receiving ART at the NHTD	1074	86.4
Temporarily received ART at another hospital	116	9.3
N/A	53	4.3
Change of ART regimen at the other hospital (among those who received ART at another hospital)		
Changed	8	6.9
No change	99	85.3
N/A	9	7.8
Discontinuation of ART because of COVID-19		
Not discontinued	1187	95.5
Discontinued	13	1.1
N/A	43	3.5
Duration of ART discontinuation (among those who discontinued ART)		
< 30 days	4	30.8
30 days	4	30.8
60 days	2	15.4
N/A	3	23.1
Support for continuing ART and HIV care during COVID-19		
Not received	206	16.6
Received	963	77.5
N/A	74	6.0
Source of support (among those who received support)^a^		
Government agency (e.g., VAAC)	145	15.1
NHTD health care staff members	903	93.8
Other PLHIV	81	8.4
Other	43	4.5
**Economic impact**		
Change in job status because of COVID-19		
No change	942	75.8
Lost jobs	174	14.0
Started working	11	0.9
Changed jobs	56	4.5
Changed job status, but no detail provided	26	2.9
N/A	34	2.7
Change in household income because of COVID-19		
No change	894	71.9
Increased	10	0.8
Decreased	251	20.2
Change in income, but no detail provided	56	4.5
N/A	32	2.6
Household income before COVID-19 (among those with a reduction in household income)		
Median (IQR), VND	8,000,000 (5,000,000 to 10,000,000)	
Amount of change in household income (among those with a reduction in household income)		
Median (IQR), VND	− 3,000,000 (− 5,000,000 to − 2,000,000)	
**Risky health behaviors**		
Change in alcohol consumption (compared with the amount before COVID-19)		
Non-drinker or no change	1009	81.2
Decreased	199	16.0
Increased	7	0.6
N/A	28	2.3
Change in drug use (compared with the amount before COVID-19)		
Non-drug user	1207	97.1
No change	1	0.1
Decreased	9	0.7
Increased	0	0.0
N/A	26	2.1
Change in condom use (compared with usage before COVID-19)		
No change	940	75.6
Decreased	16	1.3
Increased	56	4.5
N/A	231	18.6
Change in the number of sex partners (compared with the number before COVID-19)		
No change	848	68.2
Increased	22	1.8
Decreased	66	5.3
N/A	307	24.7
**Mental health**		
General distress (DASS-21-V)		
Mean ± SD	9.2 (18.2)	
Distressed (DASS-21-V ≥ 34)	95	7.6
Not distressed (DASS-21-V < 34)	1000	80.5
N/A	148	11.9

*COVID-19* Coronavirus disease 2019, *N/A* not available, *ART* antiretroviral therapy, *VAAC* Ministry of Health, Vietnam Administration of HIV/AIDS Control, *NHTD* National Hospital for Tropical Diseases, *PLHIV* people living with HIV, *IQR* interquartile range, *VND* Vietnamese dong, *DASS-21-V* the Vietnamese version of the Depression, Anxiety, and Stress Scale-21

^a^Multiple responses were allowed

^b^Social contact included non-face-to-face contacts through email, phone, or social networking services

### HIV Continuum of Care

In total, 116 participants (9.3%) were referred to other HIV outpatient clinics during the first COVID-19 outbreak. Among these patients, eight changed ART regimens at the other outpatient clinic. Thirteen patients (1.1%) reported that they discontinued ART because the outbreak caused them to run out of medicine; four participants reported that they discontinued ART for 30 days, and two reported discontinuing ART for 60 days (Table 3). A total of 963 participants (77.5%) received social support for continuing HIV care and treatment during the first COVID-19 outbreak; 93.8% of these participants received this support from NHTD health care staff members, 15.1% from government agencies (e.g., the Vietnam Administration of HIV/AIDS Control), and 8.4% from peers (Table 3). Table 4 shows HIV viral load and loss to follow-up before and after the first COVID-19 outbreak. HIV viral load did not differ much before and after the outbreak; most participants had an undetectable HIV viral load in both periods. None of the examined impacts of COVID-19 (i.e., preventive behaviors against COVID-19, HIV continuum of care, economic security, and risky health behaviors) were associated with having a detectable HIV viral load (> 20 copies/mL) after the outbreak (Table 5). However, the loss to follow-up rate was lower after the outbreak (1.4%, *n* = 18) than before the outbreak (1.8%, *n* = 24) (Table 4).

**Table 4:** HIV viral load, loss to follow-up, and general distress before and after the COVID-19 outbreak

	Before COVID-19^a^		After COVID-19^a^	
	*n*	%	*n*	%
HIV viral load (copies/mL)				
Median (range)^b^	0 (0 to 265,000)		0 (0 to 118,000)	
< 20	1174	94.5	1137	91.5
20–199	56	4.5	99	8.0
200–999	7	0.6	2	0.2
≥ 1000	6	0.5	5	0.4
Loss to follow-up^c^				
Number of participants on the first day of follow-up	1344	100.0	1288	100.0
Number of participants lost to follow-up during a 6-month follow-up period	24	1.8	18	1.4

*COVID-19* Coronavirus disease 2019

^a^The 6-month follow-up periods before and after the COVID-19 epidemic were from June 1, 2019, to November 30, 2019, and from December 1, 2019, to May 31, 2020, respectively

^b^To calculate median HIV viral load, the results of undetectable and < 20 copies/mL were treated as 0 copies/mL and 10 copies/mL, respectively

^c^Loss to follow-up was defined as a patient missing a scheduled visit without a known reason

**Table 5:** Results of univariate logistic regression analyses: factors associated with detectable HIV viral load and general distress after the COVID-19 outbreak

	Detectable HIV viral load^a^ (> 20 copies/mL) *n* = 1243		General distress^a^ (DASS-21-V ≥ 34) *n* = 1095	
	OR (95% CI)	*p*	OR (95% CI)	*p*
**Behaviors to prevent COVID-19**				
Protective behaviors against COVID-19				
Not practiced	1.00	0.22	1.00	0.17
Practiced	0.51 (0.17–1.51)		0.47 (0.16–1.40)	
Social contact^b^ with other people during COVID-19				
No change	1.00	0.41	1.00	0.98
Decreased	0.76 (0.51–1.14)		0.98 (0.64–1.51)	
Increased	–		1.18 (0.15–9.57)	
**HIV continuum of care**				
Access to ART during COVID-19				
Continued receiving ART at the NHTD	1.00	0.35	1.00	0.02
Temporarily received ART at another hospital	0.69 (0.31–1.52)		2.06 (1.12–3.81)	
Discontinuation of ART because of COVID-19				
Not discontinued	1.00	0.91	1.00	0.98
Discontinued	0.89 (0.11–6.89)		–	
Support for continuing ART and HIV care during COVID-19				
Not received	1.15 (0.68–1.95)	0.60	2.21 (1.35–3.60)	< 0.01
Received	1.00		1.00	
**Economic impact**				
Change in job status because of COVID-19				
No change	1.00	0.81	1.00	< 0.0001
Lost jobs	0.66 (0.35–1.27)		3.43 (2.06–5.70)	
Started working	–		4.93 (0.97–25.0)	
Changed jobs	0.96 (0.38–2.48)		2.41 (1.03–5.60)	
Changed job status, but no detail provided	0.82 (0.19–3.52)		2.61 (0.74–9.18)	
Change in household income because of COVID-19				
No change	1.00	0.97	1.00	< 0.0001
Increased	–		5.31 (1.04–27.02)	
Decreased	1.06 (0.65–1.72)		3.32 (2.08–5.29)	
Changed income, but no detail provided	0.81 (0.28–2.28)		2.86 (1.22–6.73)	
**Risky health behaviors**				
Change in alcohol consumption (compared with the amount before COVID-19)				
Non-drinker or no change	1.00	0.54	1.00	0.06
Decreased	1.33 (0.81–2.21)		1.77 (1.06–2.95)	
Increased	–		2.96 (0.33–26.82)	
Change in drug use (compared with the amount before COVID-19)				
Non-drug user	1.00	0.37	1.00	1.00
No change	–		–	
Decreased	3.13 (0.64–15.26)		–	
Increased	–		–	
Change in condom use (compared with usage before COVID-19)				
No change	1.00	0.44	1.00	< 0.01
Decreased	0.73 (0.10–5.57)		2.20 (0.48–10.04)	
Increased	0.40 (0.10–1.69)		3.56 (1.69–7.52)	
Change in the number of sex partners (compared with the number before COVID-19)				
No change	1.00	0.72	1.00	< 0.0001
Decreased	0.82 (0.32–2.10)		3.63 (1.80–7.29)	
Increased	0.48 (0.06–3.59)		4.32 (1.50–12.41)	

*COVID-19* Coronavirus disease 2019, *OR* odds ratio, *CI* confidence interval, *DASS-21-V* The Vietnamese version of the Depression, Anxiety, and Stress Scale-21 (DASS-21), *ART* antiretroviral therapy, *NHTD* National Hospital for Tropical Diseases

– indicates that the odds ratio could not be calculated because the number of participants in the category was too small

^a^HIV viral load and DASS-21-V score were obtained in June or July 2020, after the first outbreak of COVID-19

^b^Social contact included non-face-to-face contact through email, phone, or social networking services

### Economic Impact

The economic impact of COVID-19 among the HIV-infected participants was large (Table 3). In total, 174 (14.0%) reported that they lost their jobs, and 56 (4.5%) reported a change in jobs. In addition, 251 (20.2%) reported a reduction in household income because of the outbreak. Among these participants, the median reduction in household income was 3,000,000 Vietnamese dong, representing a 37.5% median reduction in pre-outbreak household income.

### Risky Health Behaviors

There were more participants whose health behaviors improved after the COVID-19 outbreak than there were participants whose health behaviors worsened. Few participants reported an increase in alcohol consumption (0.6%) or drug use (0.0%), whereas 199 (16.0%) reported that they reduced their amount of alcohol consumption, and nine (0.7%) reported that they reduced their drug use after the outbreak. Additionally, 56 participants (4.5%) increased their frequency condom use during sex, and 66 (5.3%) reported a decrease in their number of sex partners after the first COVID-19 outbreak—these percentages were higher than those for a decrease in the frequency of condom use (1.3%) and for an increase in the number of sex partners (1.8%).

### Mental Health

The mean total DASS-21-V score was 9.2 in this population, with a 7.6% prevalence of general distress (DASS-21-V ≥ 34) (Table 3). In the univariate logistic regression model, in contrast to the result that no COVID-19-related factors were associated with HIV viral load after the outbreak, those who temporarily received ART at another hospital during the first outbreak (OR 2.06, 95% CI 1.12–3.81; vs. those who continued to receive ART at the NHTD), those who did not receive social support (OR 2.21, 95% CI 1.35–3.60; vs. those who received social support), those who lost their jobs (OR 3.43, 95% CI 2.06–5.70) or changed jobs (OR 2.41, 95% CI 1.03–5.60; vs. those who did not experience a change in employment), those whose household income increased (OR 5.31, 95% CI 1.04–27.02), decreased (OR 3.32, 95% CI 2.08–5.29), or changed in an unknown way (OR 2.86, 95% CI 1.22–6.73; vs. those whose household income was unchanged), those who used condoms more frequently during sex (OR 3.56, 95% CI 1.69–7.52; vs. those who had no change in condom use), and those who had either decreased (OR 3.63, 95% CI 1.80–7.29) or increased (OR 4.32, 95% CI 1.50–12.41) their number of sex partners (vs. those who had no change in the number of sex partners) were more likely to have experienced general distress after the outbreak (Table 5).

## Discussion

### Incidence of SARS-CoV-2 Infection in the First COVID-19 Outbreak

In this study, we elucidated how PLHIV in Vietnam responded to and were affected by COVID-19 during the country’s first outbreak of the disease. Although most participants practiced preventive measures against COVID-19 and no one reported symptoms associated with COVID-19, the prevalence of anti-SARS-CoV-2 IgG antibodies was 0.2% at the time of this study. According to a public data source [33], the cumulative number of SARS-CoV-2 infections in the Vietnamese population of approximately 96.484 million people [34] was 558 (0.58 per 100,000 people) as of July 31, 2020. In Hanoi, the Department of Health of Hanoi reported 123 confirmed cases in the population of approximately 8.093 million [34] (1.52 per 100,000 people) as of July 31, 2020. Therefore, the positivity rate for antibodies against SARS-CoV-2 among PLHIV in our study was higher than expected, given the reported number of COVID-19 cases in Vietnam. However, we cannot conclude that this higher prevalence can be attributed to HIV infection. Currently, no evidence has demonstrated that PLHIV are more vulnerable to acquiring COVID-19. The three study participants who had anti-SARS-CoV-2 IgG antibodies did not show any special features in terms of demographic characteristics, HIV-related factors, or preventive behaviors. All three of these participants were in stable HIV condition with an HIV viral load < 20 copies/mL and a CD4 count of > 200/µl before the outbreak, and none of these participants had comorbidities. Additionally, all three of these participants reported practicing preventive measures against COVID-19 during the outbreak. The lack of data on anti-SARS-CoV-2 IgG antibodies among the general Vietnamese population precluded us from directly comparing the prevalence of antibodies in the HIV-positive population with that in the general population. However, the positivity rate for such antibodies in this study may have reflected the cumulative number of SARS-CoV-2 infections in the asymptomatic population at the time. Presumably, the testing capacity for detecting SARS-CoV-2 infection in the population was not sufficient during the first outbreak, and there were a relatively large number of “hidden” people, who were infected with SARS-CoV-2 but were not reported. During the first outbreak in particular, private information such as names, addresses, and even facial photos of people infected with SARS-CoV-2 was circulated through social networking services. The fear of privacy violations may have made people hesitant to undergo testing for SARS-CoV-2 infection in Vietnam [35, 36].

### Impact of COVID-19 on the HIV Care Continuum

Threats to continuous HIV treatment during the COVID-19 pandemic have been reported in many countries, such as the United States, Italy, and China, as well as countries in sub-Saharan Africa [14, 17, 37, 38]. Likewise, in this study in Vietnam, numerous participants (*n* = 116, 9.3%) were referred to other HIV outpatient clinics for ART during the outbreak. However, the present study found that ART interruption rates were low (1.1%), loss to follow-up rates dropped from 1.8% before the outbreak to 1.4% after the outbreak, and most patients maintained a well-controlled HIV viral load during the outbreak. The successful maintenance of HIV treatment during the first COVID-19 outbreak in this study setting may be explained by the early activation of effective responses to COVID-19 at all HIV service levels, as well as close collaboration and coordination among the levels. For example, at the government level, beginning in the very early stages of the COVID-19 pandemic, the Vietnam Administration of HIV/AIDS Control issued various guidelines to ensure the continuation of HIV care and treatment. These guidelines allowed multi-month ART refills for patients on ART. The standard referral requirements were also eased to allow clients temporary access to alternative HIV clinics for ART refills. Moreover, although HIV services in Vietnam have been transitioning into public health services that are paid for using social health insurance, HIV patients, including those facing economic disadvantage because of COVID-19, were able to receive ART free of charge in most ART facilities during the study period. This situation may also have contributed to the maintenance of HIV treatment during the outbreak. At the provincial and community levels, information on both COVID-19 and HIV was collectively managed by the provinces, and PLHIV peer networks played a role in connecting PLHIV to ART refills in communities [39]. At the hospital level, at the NHTD, the HIV outpatient clinic contacted all registered patients during the outbreak and provided telephone counseling on health status, individual concerns, and the prevention of COVID-19. For patients who needed ART refills during the hospital lockdown and for those who had difficulty visiting the NHTD because of traffic controls, the NHTD introduced alternative clinics located near patients’ residences and arranged appointments for them. To ensure a smooth referral process for these patients, close communication was maintained with the clinics through letters, telephone calls, and text messages. The NHTD’s HIV outpatient clinic also maintained its own smartphone, which it used to set up a social networking service account to promote communication between the hospital and outpatients. The smartphone was managed by a nurse in a rotating system and accepted calls and messages from patients 24 h a day. Although this finding cannot be generalized to other facilities, the services offered at the NHTD helped health care staff members to provide support that was tailored to each patient’s needs. Finally, patients cooperated well with authorities and service providers to continue their HIV treatment. Because most study participants had been on ART for a long time, they may have had a good understanding of the importance of continuing to take ART. In addition, the cultural hierarchy and the trust relationships between patients and authorities/health care providers may have enabled patients with HIV to accept temporary hospital transfers and to continue their medication.

### Socio-Behavioral Impact of COVID-19

Although HIV treatment was successfully maintained during the first COVID-19 outbreak in the studied population, COVID-19 had other substantial socio-behavioral impacts among PLHIV. In particular, the economic impact was already enormous at the time of the survey; nearly one-fifth of the participants had lost their jobs or changed jobs, and more than one-fifth had experienced a reduction in household income, with a median reduction of 37.5%. In Vietnam, the informal sector is a predominant part of the economy. In 2019, approximately half of the working population over the age of 15 years was working in this sector (e.g., through self-employment or family businesses) [40]. Informal laborers work without labor contracts and lack the basic benefits usually provided by a formal job, including unemployment insurance. Therefore, previous research in Vietnam has indicated that workers in this sector are among the most vulnerable to the impact of the COVID-19 pandemic [41, 42]. Many participants in the present study worked in the informal economy, with only 33.6% of the study cohort reporting that they were full-time employees before COVID-19. Additionally, 174 participants (14.0%) reported that they lost their jobs because of COVID-19, which was a far higher rate than the 2.4 million (4.6%) out of 51.8 million employed workers in Vietnam who lost their jobs in the second quarter of 2020 [43]. PLHIV, who are already likely to be suffering from poverty and unemployment, tend to be more economically affected by COVID-19 compared with the general population. Nevertheless, there were more participants in our study who reported improvement in their health behaviors because of COVID-19 than there were those who reported that their health behaviors worsened. These findings align with previous work in the United States and Australia indicating declining levels of drug use and engagement in risky sexual behaviors because of social distancing and stay-at-home orders to combat COVID-19 [14, 44–46]. However, it is uncertain whether such behavioral changes can be sustained in the long term. Notably, a previous study reported that, for sexual minority men in the United States, although illegal drug use and engagement in risky sexual behaviors declined during COVID-19, the association between these two factors became stronger compared with the pre-COVID-19 period [46].

### Socio-behavioral Impact of COVID-19 and Mental Health

Although none of the above-mentioned impacts of COVID-19 on the HIV care continuum, economic security, and health behaviors during the first COVID-19 outbreak showed an association with HIV viral load after the outbreak, these factors were significantly associated with mental health after the outbreak. Overall, 7.6% of the participants had general distress, as measured by the DASS-21-V. Although a previous study suggested an optimal cut-off score of 34 for detecting general distress in the Vietnamese population [31], no study has reported the prevalence of general distress using that cut-off score. Therefore, we were unable to compare the percentage observed in this study for general distress with findings for the pre-COVID-19 period or among other populations in Vietnam. However, importantly, this study found that those who temporarily visited other clinics to receive ART during the outbreak, those who did not receive social support for continuing ART and HIV care, those who lost or changed their jobs, and those who experienced a change in household income because of COVID-19 were statistically more likely to experience general distress after the outbreak. Given that COVID-19 may make it difficult for PLHIV to interact directly with health care providers, which is of particular importance for mental health care for this population, our findings provide potentially useful insight for future interventions to improve the mental health and well-being of PLHIV during this difficult time. For example, our findings suggest that social support (i.e., informational and emotional support) should be improved, especially for patients who are transferred to other hospitals because of COVID-19. HIV infection is often stigmatized [47–50], and health care providers are sometimes the only source of support for PLHIV outside their families. Further assessment is needed to understand effective social support interventions that can reduce the psychological burden of hospital transfer. Moreover, financial assistance and employment support for vulnerable patients with HIV should be strengthened at all levels. The Vietnamese government has introduced a 2.7 billion US dollar relief fund to support all workers affected by the COVID-19 pandemic in Vietnam [51]. However, it is unclear how much of this financial assistance has reached the most vulnerable people, such as those who have been laid off from informal-sector jobs or day laborers.

Increasing the frequency of condom use during sex and increasing or decreasing the number of sex partners were also positively associated with general distress after the outbreak. PLHIV’s behavioral changes in terms of substance use and sexual behaviors should be closely and continuously monitored, especially during the pandemic. Even if health behaviors are temporarily improved through the response to social distancing orders, this change could amplify psychological distress and, in turn, lead to an increase in risky health behaviors [44, 52, 53]. Mental health problems such as depression are associated with poorer adherence to ART [54, 55] and with poor HIV outcomes [56], which may also lead to negative COVID-19 outcomes [9–11]. Therefore, there is a strong need to address the possible risk factors for mental distress discussed above, not only to maintain this population’s mental health, but also to prevent both HIV and COVID-19 infection and progression.

To our knowledge, this is the first study to investigate the incidence of SARS-CoV-2 infection and the impacts of COVID-19 on the HIV care continuum, economic security, risky health behaviors, and mental health using a large cohort of PLHIV receiving ART in Vietnam. By using a long-standing cohort of patients with HIV from the pre-COVID period, we were able to contrast HIV viral load and treatment adherence before and after the COVID-19 outbreak. However, this study had several limitations. First, we adopted a cross-sectional design, so we were unable to determine any causal relationships. Second, our study site was a large HIV outpatient referral clinic in Hanoi, and most participants had been receiving ART at the NHTD for a long time, which may limit the generalizability of our findings. Moreover, this study had no control group such as HIV-negative participants, which precluded us from having more detailed insight into our findings, especially regarding the positivity rate for antibodies against SARS-CoV-2 among PLHIV. Furthermore, the impact of COVID-19 changes over time. Our findings from the first COVID-19 outbreak may not be applicable to other periods. Therefore, continuous assessment is required to monitor the impact of COVID-19. Finally, although our study provides crucial insight for effective approaches to mitigating the negative effects of COVID-19, future studies should include more rigorous evaluations of social support (e.g., support content), economic security (e.g., employment status and financial assistance), and risky health behaviors (e.g., actual change in drug use and risky sexual behaviors).

In conclusion, HIV treatment was successfully maintained without ART interruption, even in a hospital that experienced a facility lockdown during the first COVID-19 outbreak in Vietnam. However, COVID-19 substantially affected economic security and risky health behaviors among PLHIV, and these impacts may have amplified psychological stress in this population. Continuous monitoring of the impact of COVID-19 on this vulnerable population and efforts to mitigate the negative effects at all levels are strongly needed.

## Supplementary Information

Below is the link to the electronic supplementary material.

Supplementary file1 (DOCX 14 kb)
