# Supplementary material for: Evaluation of SARS-CoV-2 Antibodies and the Impact of COVID-19 on the HIV Care Continuum, Economic Security, Risky Health Behaviors, and Mental Health Among HIV-Infected Individuals in Vietnam
Source: AIDS Behav. 2021 Sep 27;26(4):1095–109. doi: 10.1007/s10461-021-03464-w (PMC8475817; doi:10.1007/s10461-021-03464-w)
Supplement: Supplementary file 1 — Supplementary file1 (DOCX 14 kb) [file 10461_2021_3464_MOESM1_ESM.docx]

Supplementary Table 1. Characteristics of three cases with positive anti-SARS-CoV-2 IgG antibody test results

|  | **Case 1** | **Case 2** | **Case 3** |
| --- | --- | --- | --- |
| **Sex** | Male | Male | Male |
| **Age (years)** | 37 | 40 | 54 |
| **Symptoms of COVID-19** | None | None | None |
| **Marital status** | Married | N/A | Married |
| **Educational attainment^a^** | High | N/A | High |
| **History of IDU** | No | Yes | No |
| **Body mass index (kg/m^2^)** | 23.1 | 18.9 | 24.9 |
| **Time since ART initiation (years)** | 7 | 7 | 6 |
| **ART regimen at the time of testing** | TDF/3TC/DTG | TDF/3TC/DTG | TDF/3TC/DTG |
| **HIV viral load (copies/mL) before the outbreak** | < 20 | Undetectable | Undetectable |
| **CD4 count (/µl) before the outbreak** | 556 | 256 | 713 |
| **Comorbidities^b^** | None | None | None |
| **Protective behaviors against COVID-19** | Yes | Yes | Yes |
| **Social contact with other people during the first COVID-19 outbreak** | Decreased | Decreased | No change |

SARS-CoV-2: severe acute respiratory syndrome coronavirus 2; COVID-19: Coronavirus disease 2019; IDU: injection drug use; ART: antiretroviral therapy; TDF: tenofovir disoproxil fumarate; 3TC: lamivudine; DTG: dolutegravir

^a^Educational attainment: low—never attended school, primary school, or junior high school; middle—high school; high—vocational school/college or university

^b^Comorbidities included hypertension, cardiovascular disease, diabetes, chronic kidney disease, chronic liver disease, and chronic respiratory disease.
